# A Novel Method of Local Anesthesia for Optical Internal Urethrotomy

**DOI:** 10.7759/cureus.6043

**Published:** 2019-10-31

**Authors:** Danny Darlington

**Affiliations:** 1 Urology, Pondicherry Institute of Medical Sciences, Pondicherry, IND

**Keywords:** internal urethrotomy, local anesthesia, stricture urethra

## Abstract

Direct vision internal urethrotomy (DVIU) is the treatment of choice for short anterior urethral strictures. It is performed under spinal or general anesthesia although it can also be done under local anesthesia. We describe a novel method of local anesthesia for internal urethrotomy. The technique described is feasible in morbid patients who are deemed unfit for spinal or general anesthesia and achieves good analgesia.

## Introduction

Direct vision internal urethrotomy (DVIU) is the treatment of choice for short anterior urethral strictures, and it generally requires spinal or general anesthesia [1-2]. However, there are several reports of DVIU being performed under local anesthesia in unfit patients [3]. Although there are different methods of local anesthesia for this endoscopic procedure, none has been proven superior in terms of achieving analgesia. The author describes a simple and cost-effective method of local anesthesia for DVIU.

## Technical report

**Figure 1:**
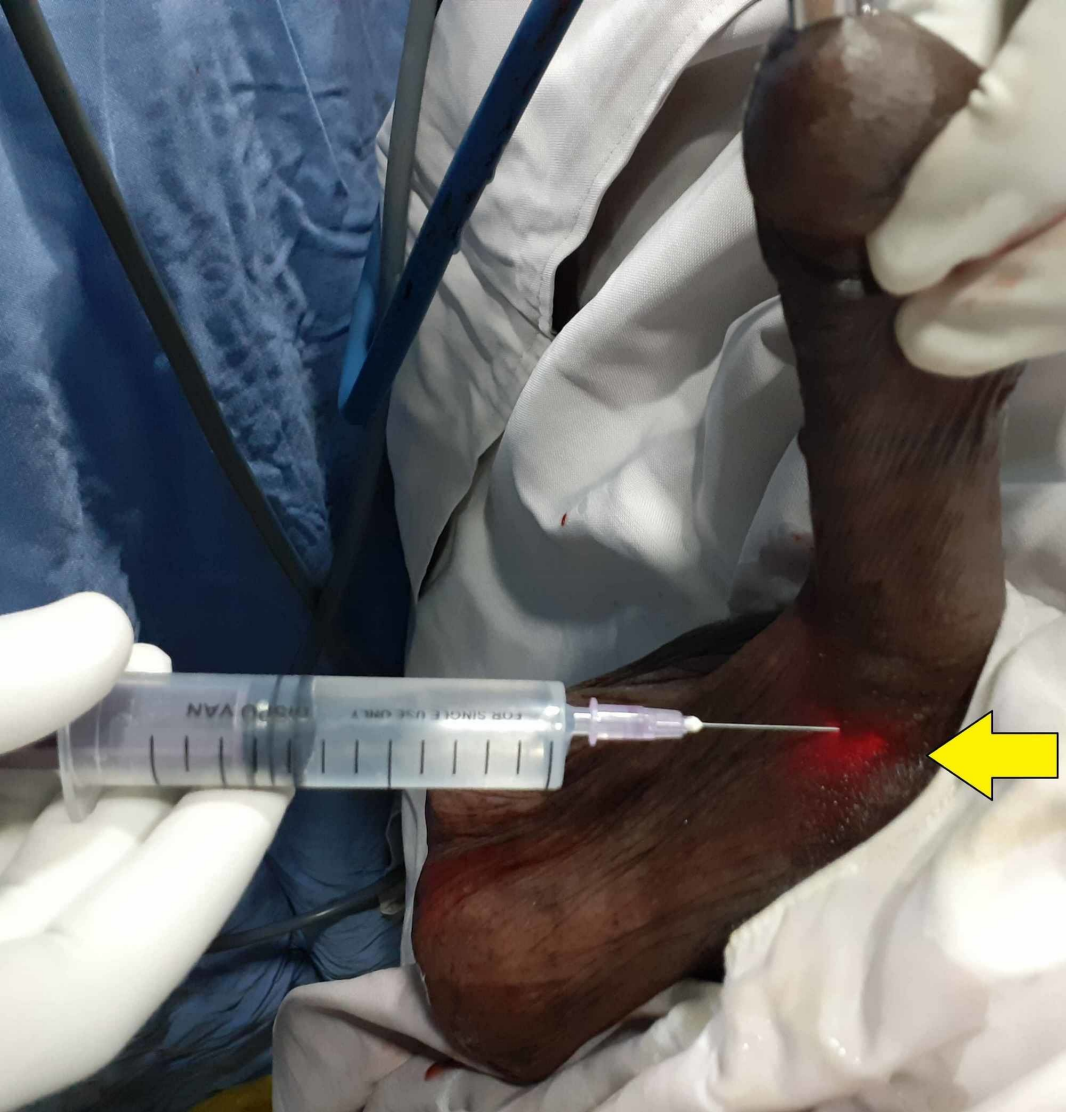
Photograph showing the local anesthetic being injected subcutaneously at the ventral midline position using the trans-illumination (arrow) as a guide to the surgeon

**Figure 2:**
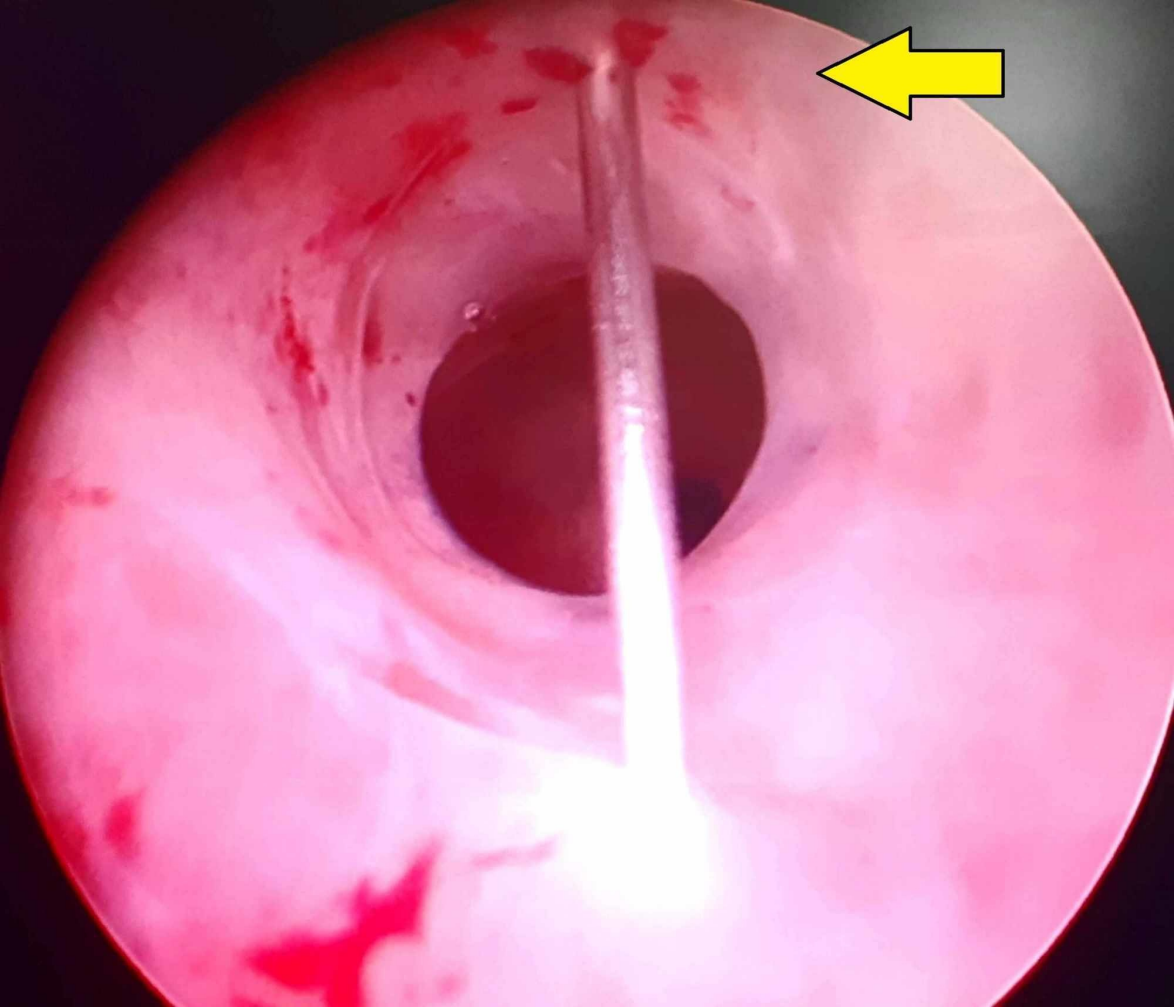
Endoscopic photograph showing the needle entering the urethral lumen at the six o’clock position and local anesthetic being injected at the 12 o’clock position of the urethra (arrow)

The patient is placed in the lithotomy position and anesthetic gel is introduced into the urethra. After 10 minutes, a diagnostic urethrocystoscopy is performed. The cystoscope is held in position at the narrowed segment of the urethra. At this step, the author recommends a series of two methods to anesthetize the urethra. One method is to inject local anesthetic solution subcutaneously into the peri-urethral region at the three and nine o’clock positions. This can be achieved by puncturing the ventral midline using a 24 g needle or a spinal needle and changing the direction of the needle thereafter to the three and nine o’clock positions followed by infiltration of the agent (Figure 1). The needle is not removed from the skin, as it is to be used for subsequent injection into the urethral lumen. The exact location for the injection can be determined by the trans-illumination of penile or perineal skin by the cystoscope held at the level of the stricture. As the entire anterior urethra is accessible subcutaneously, this method can be used for short segment strictures anywhere along the anterior urethra. Since most of the urethrotomy is performed at the twelve o’clock position and the urethrotomy must be extended deep into scar tissue proximal and distal to the stricture, deeper infiltration is needed to achieve anesthesia. Using the trans-illumination of the scope as the guide, the same needle is used to enter the urethral lumen from the six o’clock position, being viewed by the endoscope. The needle after entering the lumen is used to inject a local anesthetic into the stricture at the twelve o’clock position proximally and distally under endoscopic guidance (Figure 2). Local anesthetic can be injected similarly in other proposed sites of urethrotomy as and when needed. After the injections, a guidewire is passed up into the bladder. The cystoscope is exchanged for an internal urethrotomy sheath and cold knife urethrotomy is performed followed by insertion of the urethral catheter.

The author has performed 55 cases of DVIU using this novel method of local anesthesia successfully. The analgesia achieved is satisfactory, with a mean visual analog score of 2 out of 10 in 55 patients. There was no untoward effect like conversion to another anesthesia or need for sedation. As in any local anesthesia, the initial injections are painful, which can be reduced by using small-bore needles.

## Discussion

DVIU is an endoscopic procedure ideally suited for the treatment of short anterior urethral strictures less than 1.5 cm in length without any spongiofibrosis. Although the risk of recurrence approaches 40% for strictures shorter than 2 cm, DVIU continues to be performed by urologists [4]. The main advantages include the ability to perform it as a daycare procedure, less pain, early recovery, and short duration of hospital stay. Another appealing advantage is that it can be performed even in surgically unfit patients under local anesthesia in minimal resource settings [5].

Although DVIU is commonly performed under spinal or general anesthesia, local anesthesia can be employed in unfit patients. Different techniques of local anesthesia are described in the literature for DVIU such as intra-urethral lignocaine, intracorpus spongiosal block, and transperineal urethrosphincteric block [6-9]. Except for the transperineal block, all other methods usually require supplementary sedation [10]. The randomized controlled trial conducted by Biswal et al. concluded that intraurethral lignocaine had a higher failure rate as compared to the spongiosal block [11]. Our technique described herein achieves acceptable analgesia without the need for any sedation.

The anatomic basis of this technique is the blockade of cavernosal nerves, which usually lie at the three and nine o’clock positions in the proximal urethra. The intraurethral anesthetic gel anesthetizes the urethral submucosal plexus of nerves all along the urethra. Direct injection at twelve o’clock into the stricture additionally blocks the cavernosal nerves in the distal urethra, which are located at the one and eleven o’clock positions [12].

One of the caveats of local anesthesia is pain intolerance and the need for sedation and other modes of anesthesia. However, our method had lower sedation requirements as compared to other methods of local anesthesia. During the local anesthetic technique, the slightest pain will make the patient apprehensive and pain intolerant. Hence, utmost care must be exercised during intraurethral lignocaine instillation. We generally inject 5 ml of anesthetic gel into the fossa navicularis and wait for five minutes before proceeding to inject 20 ml of anesthetic gel into the urethral lumen. Another useful tip is to ask the patient to relax his sphincter while injecting the anesthetic gel. Overall, good communication between the surgeon and the patient on the table is necessary for a successful block.

The advantage of our technique is its reproducibility, cost-effectiveness, and high success rates, thereby enabling more urologists to perform internal urethrotomy in unfit patients. It also avoids the complications of regional anesthesia [13]. Although the injection of an anesthetic agent into the stricture can be done by using an endoscopic injection needle passed through a cystoscope, the associated high cost and limited availability of the endoscopic injection needle make it less attractive in limited-resource settings. Our technique is simple and cheap, consuming only a spinal needle or a hypodermic needle. Another advantage is it involves only a skin prick thereby mitigating the pain associated with repeated needle pricks. The main disadvantage of our technique is the theoretical risk of causing a peri-urethral abscess and injury to the corpora spongiosum causing hematuria. However, the author has not encountered such complications and further studies must be carried out to fully assess the incidence of these complications. With limited to no previous literature available to support this novel technique, further randomized controlled trials are needed to ascertain or refute the role of our technique in the endoscopic management of urethral strictures.

## Conclusions

DVIU is a commonly performed endoscopic procedure for the treatment of short segment urethral strictures. It can be performed under local anesthesia in selected patients. The technique described by the author is a novel method of local anesthesia and can be used by urologists to perform DVIU in surgically unfit patients. It can be performed in resource-poor settings and, therefore, encourages more urologists to adapt local anesthesia. This relatively new technique has not been compared head to head with other techniques and, hence, further studies are needed before it can be incorporated into clinical practice.
